# Nervous Diseases

**Published:** 1905-11-04

**Authors:** 


### NERVOUS DISEASES.

**Myasthenia Gravis.**—The disease known as myasthenia gravis, or asthenic bulbar paralysis, is rare. It has most frequently been observed in young adults. It consists in an affection of the voluntary muscles, chiefly those of the head, face, and neck, though those of the limbs and trunk may be involved. The muscles rarely become atrophied, and exhibit no tremors, fibrillary twitchings, or spasm, but their function is perverted as they become rapidly paralysed by use, although again restored by rest. The electrical reactions also are peculiar, for while the response to galvanism is unaltered, that to faradism, normal on the first application, is rapidly diminished, and finally ceases. The tired muscle, however, again responds after a rest. This reaction is peculiar to the disease, and is known as the *myasthenic reaction*. The *symptoms* most commonly observed are those due to involvement of the muscles about the head and neck, and consist in ptosis, diplopia, difficulty in swallowing, immobility of the face, inability to support the head, and difficulty of articulation. These symptoms may only appear after brief exertion, thus the eyelids can be raised, but soon droop again, a sentence may be started, but the voice rapidly tires, and pronunciation becomes confused. The indistinctness of speech is not due to any mental hebetude. When all the muscles of the body are affected the patient may be unable to walk, sit up, or raise the arms. Not infrequently the respiratory muscles are involved, and he is then liable to dangerous attacks of dyspnœa. Death is frequent, and may result from respiratory paralysis, as in a case recently reported by C. W. Burr.[7] Many patients live for years, or recover, but the case referred to, that of a strong vigorous young man of thirty, was fatal in two months. As usual, control of the sphincters was maintained throughout, there were no sensory symptoms, and the deep reflexes were not exaggerated. The picture of the disease is in some respects similar to that presented by progressive bulbar palsy, from which, however, it is distinguished by the absence of muscular wasting and tremor, the characteristic electrical reaction, and the fatigue reaction.

The pathology of the disease is obscure. Naturally the cause has been sought in the nervous system, but no definite or constant organic lesions have been found, neither has the theory of a toxæmia of the lower neurones been substantiated, and many autopsies have been negative in result.

Recent work has shown that the cause may lie altogether outside the nervous system. In a good many cases the thymus gland is persistent or present in a diseased condition, and infiltration of the muscles with lymphoid cells has been observed. Since Weigert, in 1901, suggested a possible connection between a persistent thymus, lymphoid infiltration of muscles, and myasthenia gravis, several cases have been published by Link, Haedelmoser, Hun, Goldflam, and others, and Farquhar Buzzard [8] has recently examined five cases post-mortem. In three of these no naked eye changes were found. Of the remaining two, one, a woman of 29, showed a persistent thymus weighing 41 grams and of characteristic lobar shape. On microscopic examination a deficiency of the eosinophile cells, so abundant in the thymus of children, was observed. In the second case a cystic neoplasm was found occupying the position of the gland, whilst in the remaining three the thymus remnants were not abnormal. In all five cases groups of cells like lymphocytes were found between the fibres of the muscles and the cells of the glandular organs. These "lymphorrhages," as Buzzard terms them, resembled capillary hæmorrhages, in which white cells had replaced red. Buzzard justly considers the finding of these groups of lymph cells in five consecutive cases as hopeful in establishing a definite morbid anatomy for the disease, but in the absence of blood changes or general lymphatic disease he cannot explain their occurrence. It is quite likely the presence of these cells may have been overlooked in many earlier autopsies. The absence of thymus tumour or enlargement in three of the five cases suggests that the persistence of this gland may be accessory only, and not the cause of the lymphoid deposits, but the frequent association of the two is at least significant, and future researches may reveal a close interdependence between them.

[7] Jour. of Nerv. and Ment. Dis., March, 1905. [8] Brit. Med. Jour., May 20, p. 1092.

*(To be continued.)*

